# Random auxetics from buckling fibre networks

**DOI:** 10.1038/s41467-019-12757-7

**Published:** 2019-10-25

**Authors:** S. Domaschke, A. Morel, G. Fortunato, A. E. Ehret

**Affiliations:** 1 Empa, Swiss Federal Laboratories for Materials Science and Technology, Experimental Continuum Mechanics, 8600 Dübendorf, Switzerland; 2 ETH Zurich, Institute for Mechanical Systems, 8092 Zürich, Switzerland; 3 Empa, Swiss Federal Laboratories for Materials Science and Technology, Laboratory for Biomimetic Membranes and Textiles, 9014 St. Gallen, Switzerland

**Keywords:** Metamaterials, Polymers, Mechanical properties

## Abstract

Auxetic materials have gained increasing interest in the last decades, fostered by auspicious applications in various fields. While the design of new auxetics has largely focused on meta-materials with deterministic, periodically arranged structures, we show here by theoretical and numerical analysis that pronounced auxetic behaviour with negative Poisson’s ratios of very large magnitude can occur in random fibre networks with slender, reasonably straight fibre segments that buckle and deflect. We further demonstrate in experiments that such auxetic fibre networks, which increase their thickness by an order of magnitude and more than quintuple their volume when moderately extended, can be produced by electrospinning. Our results thus augment the class of auxetics by a large group of straightforwardly fabricable meta-materials with stochastic microstructure.

## Abstract (web summary)

Auxetic materials have usually been developed with periodically arrange structures. Here, the authors electrospin fibre networks, and show both theoretically and experimentally that these random structures have pronounced auxetic behaviour with negative out-of-plane Poisson’s ratio of large magnitude.

## Introduction

Materials with negative Poisson’s ratios do not contract but expand transverse to the direction of extension when stretched. They were termed^1^ auxetic and the utilisation of their unusual behaviour has been attributed great potential in various technical fields, including medical, protective, sensor and filter applications^2^. The evidence for negative Poisson’s ratios in some directions of crystals dates back to the 19th century^3,4^, but the interest in the phenomenon of auxeticity has strongly increased^2^ since structures with notably negative Poisson’s ratios were reported^5,6^, and auxetic polymeric foams^7^ and sheets^8^ were demonstrated in the 1980s. While the origin of auxetic behaviour can be associated with different physical processes^9,10^, the majority of macroscopically auxetic materials owe this peculiar behaviour to mechanisms occurring at lower length scales and governed by particular microstructures. Therefore, by taking control over the microstructure to achieve negative Poisson’s ratios in man-made materials, a wide range of auxetic meta-materials has been designed (cf. refs. ^2,10,11^), and their realisation particularly profits from the recent advances in additive manufacturing techniques^12^. The vast majority of these designs rely on highly deterministic, periodic lattices of repeating unit cells, whose auxetic behaviour often follows from purely geometric design principles^13^. Notwithstanding, auxetic characteristics can occur in less ordered systems such as sheets with a random pattern of cuts^14^, pruned planar random networks^15^, crumbled sheets and foils^16,17^, and self-entangled, very long single wires^18^. Eventually, negative out-of-plane Poisson’s ratios were observed for few quasi-planar fibrous materials, such as paper^19^, certain non-woven fabrics^20^ or sintered stainless steel mats^21^, and the effect was attributed to a reversal of structural alterations induced during the manufacturing process, in particular, to the straightening of curved fibres^22,23^, or to the erection of tilted fibre columns^24^.

Here, we report on a pronounced auxetic effect occurring in random fibre network structures with predominantly planar fibre disposition. Different from other auxetic mechanisms in such fibrous materials reported before, this behaviour is caused by the out-of-plane buckling of transversely oriented fibre segments with high aspect ratio. Specifically, our theoretical analysis shows that the corresponding out-of-plane Poisson’s ratios may easily take values way below −100. What is more, we demonstrate that a particular type of such super-auxetic networks, consisting of fibres with few hundred nanometres in diameter, can be produced by the process of electrospinning. In line with the analytical model, the experimental analysis of these auxetic nanofibrous non-woven membranes reveals negative Poisson’s ratios with very large magnitude and a considerable increase in membrane thickness that is not compensated by the lateral contraction, so that the longitudinal extension is accompanied by a strong, several-fold increase in volume (Supplementary Movie 1). Finally, we perform multi-scale computer simulations to rationalise the observed behaviour, and thus exemplify their use as a tool to design and analyse random fibre networks as stochastic meta-materials with tailored auxetic behaviour.

## Results

### Buckling causes gigantic auxetic behaviour of fibre networks

**Fig. 1:**
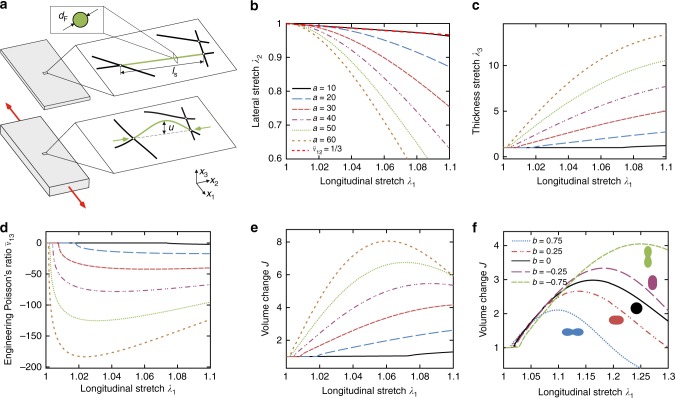
Theoretical model of the auxetic mechanism. **a** With network extension, longitudinally oriented segments reorient and subject transversal ones (green) to compressive loads, so that they buckle and deflect out of plane (${x}_{3}$) with a maximum deflection $u$. **b**, **c** Lateral in-plane (${\lambda }_{2}$) and thickness, i.e., out-of-plane (${\lambda }_{3}$) stretches vs. longitudinal stretch ${\lambda }_{1}$ for different segment aspect ratios $a={l}_{{\rm{s}}}/{d}_{{\rm{F}}}$. **d** Engineering Poisson’s ratio ${\tilde{\nu }}_{13}$ computed from (**c**). **e** Volume changes $J$ computed from (b, c). **f** Effect of initial segment orientation of segments with aspect ratio $a=20$, specified by the concentration parameter $b$ of the von-Mises distribution. Increasing values of $b$ indicate alignment with the longitudinal axis, pictograms show polar plots of the distributions

**Fig. 2:**
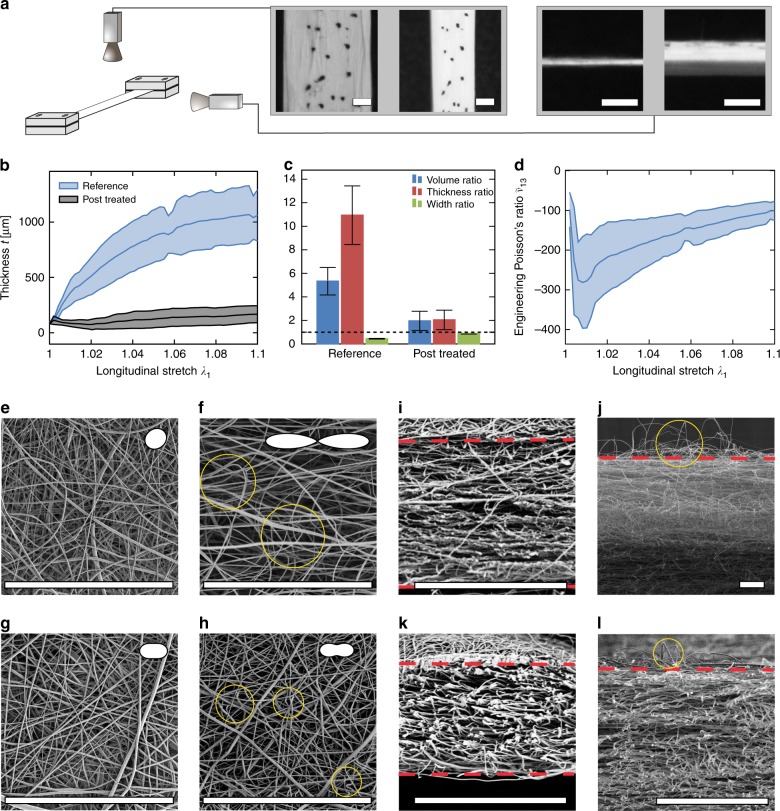
Auxetic electrospun networks. **a** Sketch of the experimental set up with representative pictures of the top and side camera at 0% strain and at a stretched state. Scale bar: 2 mm. **b** Diagram showing the evolution of the thickness for applied strain ${\lambda }_{1}$ of the reference and post-treated networks. **c** Ratios of volume, thickness and width between 10 and 0% strain show significant differences between reference and post-treated mats (*p* < 0.01, two-sided Mann–Whitney *U*-tests). **d** Engineering Poisson’s ratio ${\tilde{\nu }}_{13}$. Data in **b**, **c** and **d** are given as mean and standard deviation (shaded area, error bars) calculated from $N=\,$5 tensile tests. **e**–**l** Fibre reorientation, thickness increase and buckling segments observed in top (**e**–**h**) and side (**i**–**l**) view scanning electron microscopy of reference network at 0% (**e**, **i**) and 10% (**f**, **j**) strain, and of post-treated network at 0% (**g**, **k**) and 10% (**h**, **l**) strain (representative images). Pictograms in (**e**–**h**) show polar plots of descriptive von Mises distributions. Yellow circles point to examples of buckled segments. Larger images of networks are shown in Supplementary Fig. 4. Dashed lines indicate top and bottom edges of the mats. Scale bar in (**e**–**l**) 80 μm

**Fig. 3:**
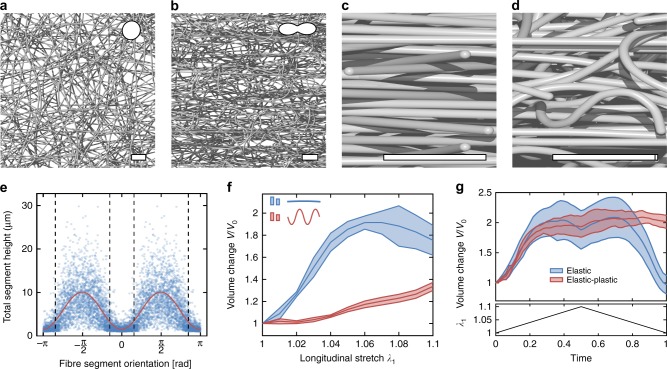
Finite element simulations. **a**, **b** Close-up top view of a simulated network with ${d}_{{\rm{F}}}=1\ \upmu$m at 0% (**a**) and 10% (**b**) strain. **c**, **d** Close-up side view of a simulated network at 0% (**c**) and 10% (**d**) strain. Scale bar in (**a**–**d**) 10 μm. Pictograms in (**a**, **b**) show polar plots of descriptive von Mises distributions. **e** Scatter plot showing the total segment height at 10% strain vs. initial segment orientation displayed from $-\pi$ to $\pi$ by use of symmetry. Trend line (red) to guide the eye, obtained by fitting the first three terms of a Fourier series. Dashed lines separate the domains, in which segment stretches are tensile or compressive, respectively, according to the affine model with aspect ratio $a=17.67$. **f** Volume change for nearly straight segments (sine shaped fibres with amplitude ${\alpha }_{{\rm{F}}}=7\ \upmu$m and wavelength ${\Lambda }_{{\rm{F}}}=150\ \upmu$m) and highly undulated segments (sine shaped fibres with ${\alpha }_{{\rm{F}}}=2\ \upmu$m and ${\Lambda }_{{\rm{F}}}=10\ \upmu$m) with applied stretch ${\lambda }_{1}$. **g** Volume change when loading and unloading a network with elastic (blue) and elastic-plastic (red) fibres, respectively, (${\alpha }_{{\rm{F}}}=7\ \upmu$m, ${\Lambda }_{{\rm{F}}}=150\ \upmu$m). The lower diagram shows the applied stretch ${\lambda }_{1}$ over normalised time. Data in (**f**, **g**) are averages of *N* = 3 network realisations and given as mean and standard deviation (shaded area)

When a network of slender fibre segments is uniaxially extended, some fibre segments realign with the direction of elongation, and the network undergoes lateral contraction (cf. ref. ^25^). As a consequence, transverse fibre segments are subjected to compressive loads and, eventually, buckle^26,27^. We hypothesised that the deflecting buckled segments drastically increase the distance that they span out-of-plane, and that the network expands through the collective response of many fibres. In order to illustrate and study this behaviour qualitatively, an affine structural modelling approach^28^ (“Methods”) was employed to compute the kinematic responses for a fibre network with different aspect ratios $a={l}_{{\rm{s}}}/{d}_{{\rm{F}}}$. The fibre segments with length ${l}_{{\rm{s}}}$ and diameter ${d}_{{\rm{F}}}$ (Fig.1a) were modelled as initially co-planar with isotropic orientation distribution within the plane and with linear elastic properties. When the critical buckling load of an Euler elastic beam is reached, they deflect by an amount $u$ calculated from a post-buckling analysis^29^. Similar as in plane triangulated truss-like structures^27^ the buckled segments are assumed to deflect preferably out-of-plane in a quasi-planar or layered network, due to the constraints imposed by the surrounding fibres. Therefore, the segments increase their total height from ${d}_{{\rm{F}}}$ to ${d}_{{\rm{F}}}+u$ (Fig. 1a). Averaging over all fibre segments, the response to uniaxial tensile loading with free lateral contraction was computed (Fig. 1b, c), and the stretches in width (${\lambda }_{2}$) and thickness direction (${\lambda }_{3}$) were calculated for a given longitudinal extension (${\lambda }_{1}$). Both the corresponding out-of-plane engineering and tangent Poisson’s ratios predicted by the theoretical model are strikingly negative and take values below −100 for the aspect ratios considered here (Fig. 1d, Supplementary Fig. 1). Finally, $J={\lambda }_{1}{\lambda }_{2}{\lambda }_{3}$ was calculated as a measure of network volume change and takes values up to 8 (Fig. 1e). The results demonstrate that the balance between lateral contraction and out-of-plane expansion, that governs the volume change, depends on the state of longitudinal extension (Fig. 1b, c), so that the the volume gain is characterised by a maximum (Fig. 1e). Since the deflection increases with $a$, while the critical buckling load decreases, the aspect ratio affects both the position and magnitude of this maximum as well as the onset of the auxetic behaviour, whereby lower ratios reduce and retard the effect (Fig. 1e). The sharp onset of the auxetic effect is caused by the buckling instability, and is expected to be much smoother in real networks (cf. Figs. 2, 3), due to the dispersion of $a$, imperfections, initial curvature, as well as additional bending moments and normal force components at the segment ends (cf. ref. ^30^). The large discrepancy between the tensile forces needed to extend a slender fibre and the compressive forces required to make it buckle suggests that a smaller fraction of fibres inclined towards the loading direction would be sufficient to cause the out-of-plane deflection of transversally oriented fibre segments. We therefore hypothesised that the initial fibre orientation is another determining factor that controls the auxetic effect. The in-plane orientation of the fibres was thus defined through a von-Mises distribution, where an increasing concentration parameter $b$ indicates stronger initial alignment with the axis of loading and $b=0$ represents the isotropic distribution considered before (Fig. 1e). The modelling results show that the fraction of buckled fibres at a given extension is strongly affected by their initial orientation (Supplementary Fig. 2), and that, indeed, a lower fraction of fibres oriented towards the loading direction (*b* < 0) is sufficient to elicit substantial network expansion (Fig. 1f), thus suggesting that the initial fibre distribution can be adjusted to maximise the volume gain under tension in a given range of deformation.

### Nano-fibrous auxetic membranes produced by electrospinning

Fibre networks with aspect ratios $a$ in the order expected to elicit noticeable auxetic behaviour can be produced by electrospinning. To illustrate that these materials show the forecasted effects, mats with uniform in-plane fibre distribution were electrospun from poly(L-lactide) (PLLA), a typical biodegradable polymer for biomedical applications^31^. The analysis of the electrospun membranes by scanning electron microscopy (SEM) revealed connections between fibres (Fig. 2e, Supplementary Fig. 3), which might originate in adhesion^32^ or fusion, that act as cross-links of the network, and that separate the fibres into segments. In order to reduce the aspect ratio $a$, a set of networks was treated with solvent vapour after spinning with the aim of increasing the number of cross-links between adjacent fibres^33^, thus decreasing the length of fibre segments. Strips of the untreated reference and post-treated electrospun mats were tested in uniaxial tension, while longitudinal extension, lateral contraction and thickness change were determined by means of top and side view cameras, that capture the tremendous expansion of the untreated mat (Fig. 2a). The comparison between the determined sample heights of untreated reference and post-treated samples clearly demonstrates the reduced thickening after exposure to solvent vapour (Fig. 2b). Estimates of the ratio between final and initial thickness, based on optically determined sample height at 0% and 10% extension (“Methods”), and on the mean initial thickness measured with a surface profiler (95.5 μm, 81.6 μm), take values of about eleven for the reference and two for the post-treated network (Fig. 2c). Taking into account the corresponding width ratios, this translates to a total increase of volume by factors higher than five for the reference and about two for the cross-linked network (Fig. 2c). The corresponding Poisson’s ratios for the reference network (Fig. 2d, Supplementary Fig. 1) confirm the theoretically predicted range of large, negative values. Concomitant with much less macroscopic reduction in width (Fig. 2c), SEM reveals lower segment reorientation (Fig. 2g, h) of the post-treated compared to the reference networks (Fig. 2e, f). Moreover, these images show the presence of buckled segments (Fig. 2f, h, j, l) in both cases, but with typically reduced length and deflection after post-treatment (Supplementary Fig. 4). The coincidence of this attenuated fibre deflection with the reduced auxetic behaviour of post-treated mats (Fig. 2c) provides further supporting evidence for buckling as the main mechanism that causes the large negative out-of-plane Poisson’s ratios (Fig. 2d). Altogether, the experimental results confirm that the auxetic behaviour and volumetric expansion predicted by the theoretical analysis, indeed exist as an intrinsic property of electrospun networks with appropriately slender fibre segments, and even occur with the anticipated orders of magnitude. In view of the versatility of the technology and the range of base materials that can be processed by electrospinning^34^, this opens up a wide design space for nanofibrous stochastic auxetic meta-materials.

### Exploring auxetic network design by computational analysis

Similar as for auxetic meta-materials with deterministic structures^13,35^, computational tools can help exploring the space of possible auxetic network designs, and support the rational analysis of existing ones. In particular, multiscale models allow virtual testing and thus provide access to information beyond experimental feasibility. To showcase this, we studied the effects of fibre tortuosity and inelasticity on the auxetic behaviour by means of a 3D finite element model of electrospun networks^25^, that overcomes the assumptions of affinity and prescribed buckling modes inherent to the idealised analytical model. Uniaxial tension simulations for a reference material with low fibre curvature, i.e., nearly straight segments and mean segment aspect ratio $\overline{a}$ = 17.67 qualitatively capture the in-plane fibre reorientation (Fig. 3a, b), the buckling fibres that push apart the originally layered structure out-of-plane (Fig. 3c, d), and the overall increase of volume observed in experiments (Fig. 2). Moreover, the simulations confirm that the total height of fibre segments in the stretched state correlates with their initial orientation, in particular, that longitudinal segments remain close to their diameter ${d}_{{\rm{F}}}$, whereas transversal segments tend to increase their total height several-fold (Fig. 3e). The simulations thus provide further evidence that the auxetic effect is caused by the preference for out-of-plane deflection of compressed fibre segments. In fact, disturbing this particular buckling behaviour through a modification of the fibre shape to planar sine waves with shorter wavelength, concomitant with a facilitation of in-plane deformations, reveals significantly less volume gain (Fig. 3f). While the buckling of elastic beams is reversible, the majority of polymers used in electrospinning have elasto-plastic material properties. Therefore, the auxetic deformations become at least partially permanent, and expansions remain after a load cycle, as seen in experiments (Supplementary Fig. 5) and predicted by corresponding simulations with elasto-plastic fibres (“Methods”, Fig. 3g). Hence, the computational study demonstrates that the topological and material properties of the fibres provide another means to tailor the network’s auxetic behaviour towards the characteristics desired for a particular application.

## Discussion

Despite the increasing availability of additive manufacturing techniques such as 3D printing^36^, the fabrication of auxetic meta-materials with highly controlled microstructures may remain a limiting factor in their use and development^2,37^, particularly on larger scale. In this regard, the enrichment of the auxetic class by the multifarious group of random fibre networks in general, and by the easy-to-produce electrospun membranes in particular, appears as a promising alternative route. In addition, the auxetic behaviour of stochastic fibre networks reported here is accompanied by considerable increases in network volume with extension, and since the fibres’ total volume remains practically constant, this entails a gain in porosity and thus alterations in surface to volume ratio and transport properties^34^. With regard to electrospun networks, the temporary or permanent activation of these alterations on demand thus suggests various useful applications from adjustable sieves^38^ to controllable drug-releasing carriers^39^, and scaffolds for tissue engineering with pore sizes that allow for improved cell infiltration^40^.

The here discussed networks represent structures formed by discrete and interconnected fibres. When considering the material formed by the network as a macroscopic solid instead, the largely layered disposition of fibres furnishes it with anisotropic material symmetry, such as transverse isotropy in the case where fibres are distributed randomly within the plane. Furthermore, the occurrence of buckling at the fibre segment scale provides this material with non-linear macroscopic properties. The well-known thermodynamic limits that restrict the Poisson’s ratio of a linear elastic isotropic material to the range between −1 and 0.5 do thus evidently not apply^41,42^ although, noteworthy, the analytical model used to study the auxetic effect agrees with these bounds for small deformations, with in-plane Poisson’s ratio 1/3 corresponding to a planar isotropic rari-constant material^43^ (Supplementary Discussion and Supplementary Fig. 6).

The theoretical model and multi-scale computer simulations revealed that thickness and volume expansion are elicited by beam-like fibre segments, that buckle and undergo large geometrical changes when set under compression by other fibres which realign under tension. Notwithstanding that the affine analytical model attributes the potential for such behaviour to any type of plane or multi-planar network with segments of appropriate aspect ratio, some fibre and network morphologies might favour the auxetic effect. As shown here by experiments and multiscale simulations it is, for example, particularly pronounced in electrospun networks which are composed of relatively straight and long, quasi-continuous fibres. Intriguingly, however, the asymmetry between the tension and compression responses of fibres, and the particular kinematics of their networks were also found responsible for large positive Poissons ratios ($$> $$5) and strong volume reductions in soft biological tissue membranes under tensile loads^44^. The combination of these results highlights even more the wide range of properties achievable by random network microstructures, with Poisson’s ratios far beyond the lower and upper thermodynamic limits of a linear elastic isotropic material.

## Methods

### Affine model

In the reference state, all segments were modelled as straight slender beams within the ${x}_{1},{x}_{2}$-plane, with circular cross-section of diameter ${d}_{{\rm{F}}}$ and length ${l}_{{\rm{s}}}=a\ {d}_{{\rm{F}}}$, forming an angle $\varphi$ with the ${x}_{1}$-axis. With network deformation the segment end-to-end vectors, i.e., the lines joining the end points of a segment, are assumed to transform in an affine manner, thus changing their original length from ${l}_{{\rm{s}}}$ to $r$. The segment stretch ${\lambda }_{{\rm{s}}}=r/{l}_{{\rm{s}}}$ is thus given in terms of the network’s in-plane principal stretches ${\lambda }_{1}$ and ${\lambda }_{2}$, here conveniently aligned with the ${x}_{1}$ and ${x}_{2}$-axes, respectively, such that

1 $${\lambda }_{{\rm{s}}}(\varphi ,{\lambda }_{{\rm{1}}},{\lambda }_{{\rm{2}}})=\sqrt{{\lambda }_{{\rm{1}}}^{2}{\cos }^{2}\varphi +{\lambda }_{{\rm{2}}}^{2}{\sin }^{2}\varphi }.$$

Under tension ($${\lambda }_{{\rm{s}}}\,> \,1$$), the segment response is linear elastic with Young’s modulus $$E$$. The compression response ($${\lambda }_{{\rm{s}}} \,< \,1$$) is modelled by Euler buckling of a double-clamped column^29^ with constant arc length $${l}_{{\rm{s}}}$$. Deflection is assumed to occur in $${x}_{3}$$-direction, with magnitude $$u$$ in the segment centre (Fig. 1a) when the compressive force $$F$$ reaches the critical buckling force $${F}_{{\rm{cr}}}=E{\pi }^{3}{({d}_{{\rm{F}}}/4a)}^{2}$$ at $${\lambda }_{{\rm{cr}}}=1-{(\pi /2a)}^{2}$$. Parametrising the segment along its arc length $$s$$, the post-buckled shape is obtained from the solution of the differential equation^29^ $$EI{\mathrm{d}}\theta /{\mathrm{d}}s=-Fy$$, where $$\theta$$ is the deflection angle, i.e., the angle between the tangent at $$s$$ and the end-to-end vector, $$I$$ the second moment of inertia and $$y$$ the deflection. For a given $${\theta }_{0}=\theta (s={l}_{{\rm{s}}}/4)$$$$\ F$$, $$u$$ and $${\lambda }_{{\rm{s}}}$$ calculate as (see ref. ^29^)

2 $$F=\frac{E\pi {d}_{{\rm{F}}}^{2}}{4{a}^{2}}{K}_{1}{({\theta }_{0})}^{2},\quad u=\frac{a\sin (\frac{{\theta }_{0}}{2}){d}_{{\rm{F}}}}{{K}_{1}({\theta }_{0})},\quad {\lambda }_{{\rm{s}}}=\frac{2{K}_{2}({\theta }_{0})}{{K}_{1}({\theta }_{0})}-1,$$

where ${K}_{1}({\theta }_{0})={\int }_{0}^{\pi /2}{(1-{\sin }^{2}({\theta }_{0}/2){\sin }^{2}\Phi )}^{-\frac{1}{2}}d\Phi$ and ${K}_{2}({\theta }_{0})={\int }_{0}^{\pi /2} (1-{\sin }^{2} ({\theta }_{0}/2) \times {\sin }^{2} \Phi )^{\frac{1}{2}}{\mathrm{d}}\Phi$ represent complete elliptic integrals of the first and second kind. The Eq. (2) were solved for steps of ${\theta }_{0}$ in a range ${\theta }_{0}\in [0,2\pi /3]$ and, by interpolation, the relations $u=u({\lambda }_{{\rm{s}}})$ and $F=F({\lambda }_{{\rm{s}}})$ were established. In the post-buckling state (${\lambda }_{{\rm{s}}}\le {\lambda }_{{\rm{cr}}}$), the segment spans the total height ${d}_{{\rm{F}}}+u({\lambda }_{{\rm{s}}})$, and a segment specific thickness stretch

3 $${\zeta }_{{\rm{s}}}({\lambda }_{{\rm{s}}})=\left\{\begin{array}{ll}\frac{{d}_{{\rm{F}}}\,+\,u({\lambda }_{{\rm{s}}})}{{d}_{{\rm{F}}}}&\ {\rm{if}}\ {\lambda }_{{\rm{s}}} \le {\lambda }_{{\rm{cr}}}\\ 1&\ {\rm{else}}\end{array}\right.$$

is defined. The force acting on the segment ends is then

4 $${F}_{{\rm{s}}}({\lambda }_{{\rm{s}}})=\left\{\begin{array}{ll}-F({\lambda }_{{\rm{s}}})&\ {\rm{if}}\ {\lambda }_{{\rm{s}}} \le {\lambda }_{{\rm{cr}}}\\ E{\pi} {d}_{{\rm{F}}}^{2}({\lambda }_{{\rm{s}}}-1)/4&\ {\rm{else}}\end{array}\right..$$

Following the affine structural approach^45^, the overall lateral force in ${x}_{2}$-direction is calculated by averaging over the normalised segment orientation density $p$, and set to zero to account for the uniaxial tension state

5 $$\int _{0}^{2\pi }p(\varphi ;b)\ {F}_{{\rm{s}}}({\lambda }_{{\rm{s}}}(\varphi ,{\lambda }_{{\rm{1}}},{\lambda }_{{\rm{2}}}))\frac{{\sin }^{2}\varphi }{{\lambda }_{{\rm{s}}}(\varphi ,{\lambda }_{{\rm{1}}},{\lambda }_{{\rm{2}}})}\ \mathrm{d}\varphi =0,$$

where $p(\varphi ;b)$ is given by the $\pi$-periodic, i.e. double-wrapped von-Mises distribution with concentration parameter $b$ and mean direction aligned with the axis of loading^46^. Equation (5) provides an implicit relationship between the stretches as ${\lambda }_{2}=f({\lambda }_{1})$. The thickness stretch ${\lambda }_{3}$ of the network is then obtained by averaging the segment specific thickness stretch

6 $${\lambda }_{3}=\int _{0}^{2\pi }p(\varphi ;b)\ {\zeta }_{{\rm{s}}}({\lambda }_{{\rm{s}}}(\varphi ,{\lambda }_{{\rm{1}}},{\lambda }_{{\rm{2}}}))\ \mathrm{d}\varphi ,$$

and the volume change results as $J={\lambda }_{1}{\lambda }_{2}{\lambda }_{3}$ for a given ${\lambda }_{1}$. Noteworthy, the Young’s modulus appears as a linear factor in the segment force ${F}_{{\rm{s}}}$ and does therefore not influence the solution of Eq. (5). The lateral contraction and thickness change can therefore be considered as a structural effect that, at least in the linear elastic range, is independent of the single fibre material properties. The integrals (Eqs. (5, 6)) were solved numerically with Matlab (R2016b, The MathWorks Inc., Natick, MA, USA) for increments $\Delta {\lambda }_{1}$ of ${\lambda }_{1}$, thus determining the corresponding increments $\Delta {\lambda }_{2}$, $\Delta {\lambda }_{3}$. While strictly, Poisson’s ratio is a material constant defined for small strain linear elasticity, several generalisations for large strains have been proposed (see ref. ^9^). Here, an engineering Poisson’s ratio ${\tilde{\nu }}_{13}$, defined through the Poisson function^47^, and the physically meaningful tangent Poisson’s ratio^48 ^${\nu }_{13}$

7 $${\tilde{\nu }}_{13}=\frac{1-{\lambda }_{3}}{{\lambda }_{1}-1},\quad {\nu }_{13}=-\frac{\partial \mathrm{ln}{\lambda }_{3}}{\partial \mathrm{ln}{\lambda }_{1}}\approx -\frac{{\lambda }_{1}}{{\lambda }_{3}}\frac{\Delta {\lambda }_{3}}{\Delta {\lambda }_{1}}$$

were computed (Figs. 1d, 2d, Supplementary Fig. 1).

### Electrospinning

PLLA pellets (3100HP Ingeo, Natureworks, USA) were dissolved in dichloromethane (DCM, Macron fine chemicals, Avantor, USA) to form a 10% polymer solutions (w/w). 2% (w/w) dimethylformamide (DMF, VWR chemicals, USA) and 0.015% (w/w) tetraethylammonium bromide (TEAB, Sigma Aldrich, USA) were added to increase relative permittivity and electrical conductivity, respectively. Electrospun fibre mats ($\sim$250 × 250 ${{\rm{mm}}}^{2}$) were produced on a needleless pilot plant (Nanospider, NS 1WS500U, Elmarco, Czech Republic) equipped with a spinning carriage module, in which the solution was poured. Parameters used were an applied voltage of +20/−17 kV, a reservoir speed of 480 mm/s, at a controlled relative humidity of 20%, and a distance of 220 mm between source and substrate.

### Sample preparation and analysis

Two pieces of approximately 80 × 100 mm^2^ were cut from the central region of an electrospun mat. One of these samples served as a reference whereas the other one was post-treated to increase the amount of cross-links between the fibres and thus reduce the segment length. Based on the recently reported effect of solvent vapour to weld adjacent fibres at cross-points^33^, the sample was stored in a desiccator for 180 min at room temperature together with a bowl of 40 ml of DCM to saturate the environment with solvent vapour. To avoid shrinkage, the membrane was clamped on each side. Reference sample thickness was determined in ambient condition with a surface profiler (Dektak 150, Veeco, USA) equipped with a tip of 2.5 μm height by measuring the height difference between the top membrane surface laying on the substrate and the bare substrate. It provided sample thicknesses of ${t}_{0}^{{\rm{P}}}$ = 96 $\pm$ 16 μm for the control (reference) and ${t}_{0}^{{\rm{P}}}$ = 82 $\pm$ 10 μm for the post-treated network (mean $\pm$ std, $N=$ 7 measurements, respectively). 80 × 10 mm^2^ specimens were cut from the 80 × 100 mm^2^ samples with a scalpel. A black pen (GeoCollege Pigmentliner, Aristo, Austria) was used to create several markers on the top surface of the samples to facilitate optical strain measurement by digital image analysis.

### Tensile testing

A custom tensile testing set-up equipped with two stepper motors controlling the clamp displacement was used to conduct the test. Specimens were mounted on two custom clampings, equipped with sandpaper to enhance grip and closed by two screws each, leaving a free sample length of $60\,{\rm{mm}}$. The displacement of the two clamps was controlled to obtain a nominal strain rate of $0.1 \% /{\rm{s}}$. Top- and side view images (1280 $\times$ 960 ${\mathrm{px}}^{2}$) of the deforming specimen were recorded at an acquisition rate of 2 Hz during the experiment by two cameras (GRAS-14S5C-C, Point Grey, Richmond, BC, Canada), synchronised and controlled with LabVIEW (National Instruments, Austin, Texas, USA) code. The top camera was equipped with a 55 mm lens (TEC-55, Computar, Cary, North Carolina, USA), the side camera with a 0.25$\times$ telecentric lens (${\rm{TECHSPEC}}$® GoldTL^TM^, Edmund Optics, Barrington, New Jersey, USA) providing a field of view of 33.024 $\times$ 24.768 ${{\rm{mm}}}^{2}$.

### Image-based deformation analysis

Local deformations in the central region of the specimen were determined from the acquired top-view and side-view images of the deforming specimens. Features around the superficial markers of the top-view images were tracked through the image sequences using a custom and calibrated optical flow based algorithm (see ref. ^49^), that provides the in-plane principal stretches ${\lambda }_{1},{\lambda }_{2}$, representing the length and width ratio, respectively. From the side-view images, the specimen height was averaged over a central 35 px wide rectangular (For comparison, the side-view images in Fig. 2a show a 218$\times$218 ${{\rm{px}}}^{2}$ cut-out). To this end, the grey-scale images were converted to binary with a threshold level of 0.25 in Matlab to distinguish between network and background. The number ${n}_{{\rm{w}}}$ of white pixels was counted and divided by the width of the strip. With a pixel size of $25.8\ \upmu {\rm{m}}$ the optically determined thickness was calculated by ${t}^{{\rm{opt}}}={n}_{{\rm{w}}}/35\times 25.8\ \upmu {\rm{m}}$. After mounting, the thin specimens were slack and typically slightly tilted or twisted. When the clamps moved apart, the specimens tightened and aligned within a plane, so that the side-view camera initially recorded a decrease in the projected sample height ${t}^{{\rm{opt}}}$. Making use of this, a taut reference (zero-strain) state (${\lambda }_{1}={\lambda }_{2}={\lambda }_{3}=1$) was defined post hoc from the side-view images as the instance where this characteristic decrease stopped, typically at a minimum of ${t}^{{\rm{opt}}}$. Due to the high ratio of sample width to thickness (around 100) a very small tilt of the side camera with respect to the specimen plane leads to a significant difference of the projected height measured by the side camera compared to the true thickness. Further uncertainty arises from the choice of the threshold level when converting from grey scale to black and white. The expected error was quantified by evaluating the difference $\epsilon ={t}_{0}^{{\rm{opt}}}-{t}_{0}^{{\rm{P}}}$ between the optically determined mean height ${t}_{0}^{{\rm{opt}}}$ in the reference state and that measured by the AFM-profiler ${t}_{0}^{{\rm{P}}}$. $\epsilon$ is treated as systematic error and subtracted from the height measured by the camera so that $t={t}^{{\rm{opt}}}-\epsilon$. Estimates of the ratio between final (at 10% strain) and initial thickness were calculated by dividing $t$ with the reference thickness ${t}_{0}={t}_{0}^{{\rm{P}}}$, and the volume ratio was computed as ${\lambda }_{1}{\lambda }_{2}t/{t}_{0}$. Similarly, tangent Poisson’s ratios ${\nu }_{13}$ and ${\tilde{\nu }}_{13}$ were calculated (Eq. (7)), using ${\lambda }_{3}=t/{t}_{0}$.

### Scanning electron microscopy

A custom-made tensile stage was used to stretch the membrane in order to perform SEM of the fibre network in a predefined strain state. For this purpose, rectangular specimens of 40 $\times$ 6 mm^2^ were clamped in the stage with an initial length of 25 mm and stretched to 10% strain by manually moving the clamps with a gear system. The sample was coated with a layer of gold palladium (10 nm thickness) by use of a sputter coater (Leica EM ACE600, Leica Microsystems, Germany). The stage was installed inside the SEM chamber (Hitachi s-4800, Hitachi High-Technologies Corporation, Japan) and imaging of the fibre networks was performed with acceleration voltage of 2 kV and a current flow of 10 μA.

### Finite element simulations

A finite element model^25^ specific for electrospun materials was used to investigate the auxetic effect. Briefly, a three dimensional structure of electrospun fibres, discretised with Timoshenko beam elements (Type B31), is generated through an initial simulation step (Abaqus/Explicit 2016, Dassault Systèmes Simulia Corp., Johnston, RI, USA) where fibres with prescribed material and shape properties are deposited on each other, similar to the manufacturing process in electrospinning. The driving mechanism behind the deposition is a body force whose magnitude is adjusted to obtain a desired porosity or mean segment length. The single fibres were described by the fibre diameter (${d}_{{\rm{F}}}=1\ \upmu {\rm{m}}$) and sinusoidal in-plane shape in the reference configuration, with prescribed amplitude ${\alpha }_{{\rm{F}}}$ and wavelength ${\Lambda }_{{\rm{F}}}$. Here, two sets of networks ($N=3$, respectively) with either nearly straight segments (${\alpha }_{{\rm{F}}}=7\ \upmu$m, ${\Lambda }_{{\rm{F}}}=150\ \upmu$m) or undulated segments (${\alpha }_{{\rm{F}}}=2\ \upmu$m, ${\Lambda }_{{\rm{F}}}=10\ \upmu$m) with dimensions of 300$\times$300$\times {t}_{{\rm{N}}}\ \upmu {{\rm{m}}}^{3}$ and a porosity of $\phi =0.961$ were generated (Fig. 3). The sets were characterised by thickness ${t}_{{\rm{N}}}$ and segment length $a$ of $19.14\,\pm\,0.04\ \upmu {\rm{m}}$ and 17.67$\pm 0.48\ \upmu {\rm{m}}$ (straight), and $19.59\,\pm\,0.4\ \upmu$m and $17.34\,\pm\,0.5\ \upmu$m (undulated), respectively (mean and standard deviation). The fibre material behaviour was defined by Young’s modulus ${E}_{{\rm{F}}}$ = 1500 MPa and Poisson’s ratio ${\nu }_{{\rm{F}}}$ = 0.4 in the elastic case and additionally with the hardening slope ${K}_{{\rm{F}}}^{{\rm{p}}}$ = 150 MPa and yield stress ${\sigma }_{{\rm{F}}}^{{\rm{p}}}$ = 30 MPa for the elastic-plastic case^25^ in a geometrically non-linear frame. The generated networks were used to conduct simulations of uniaxial tension experiments with Abaqus/Explicit by prescribing the elongation (${\lambda }_{1}$) of the virtual network through homogeneous displacements at two opposite cross-sections, homogeneous displacements and overall zero traction at the two lateral boundaries, and unconstrained bottom and top surfaces^25^. Simulation output, i.a. forces and displacements at the nodes of the finite element mesh were saved at constant time increments, starting with the reference frame. The current nodal positions were extracted, imported into Matlab and for each segment the total segment height was determined as the difference between maximal and minimal out-of-plane position along the segment (Fig. 3e). A compact boundary enveloping the point cloud of all nodes was computed by means of the ‘boundary’ command in Matlab, to obtain the volume $V$ taken by the virtual network sample at each time step, i.e., each increment in longitudinal stretch ${\lambda }_{1}$. Dividing by the reference volume ${V}_{0}$ obtained from the first frame, the volume change $V/{V}_{0}$ was computed (Fig. 3f, g). For visualisation of results (Fig. 3a–d), ray-tracing software (POV-Ray, Persistence of Vision Raytracer Pty. Ltd, Williamstown, VIC, Australia) was utilised.

### Segment orientation distributions

To extract fibre orientation distributions from SEM (Fig. 2e–h) and rendered simulation results (Fig. 3a, b), the multiscale principal component analysis algorithm^50^, implemented in Matlab^51^ was used. The image-based distributions were approximated by $\pi$-periodic von-Mises orientation distribution functions ${\varrho }_{{\rm{vM}}}$^46^, whose concentration parameter $b$ and mean angle ${\varphi }_{0}$ were identified by least-squares optimisation. Pictograms of the distribution were generated from polar plots $r={\varrho }_{{\rm{vM}}}(\varphi )$.

### Statistics

Experimentally determined differences in the volume, thickness and width between post-treated and untreated reference electrospun networks (Fig. 2c) were evaluated by two-sided Mann–Whitney *U*-tests with Matlab.

## Data Availability

All relevant data are available from the authors upon request, and/or are included within the main part and Supplementary Information.

## Supporting Information

Supplementary Information
Description of Additional Supplementary Files
Supplementary Movie 1
