# Supplementary material for: Random auxetics from buckling fibre networks
Source: Nat Commun. 2019 Oct 25;10:4863. doi: 10.1038/s41467-019-12757-7 (PMC6961253; doi:10.1038/s41467-019-12757-7)
Supplement: Supplementary file 1 — Supplementary Information [file 41467_2019_12757_MOESM1_ESM.pdf]

Supplementary Information

Random auxetics from buckling fibre networks

Domaschke et al.

Supplementary Discussion

Small-strain Poisson's ratios of the analytical model. The affine analytical model used to explain the basic mechanisms responsible for the pronounced auxetic behaviour of random fibre networks can be viewed as an ideal isotropic planar (2D) material in the reference state for $b = 0$. Before buckling sets in, the model represents the limit case of straight linear elastic fibres, with initially uniform orientation distribution in a plane, that transmit only axial (central) forces. Such a material is expected to feature the Poisson's ratio $\nu = 1/3$ of a plane isotropic rari-constant material[1]. In fact, plotting a line with slope -1/3 together with the λ_2 vs. λ_1 curves obtained by means of the analytical model (Fig. 1b), it is observed that this theoretical limit is met for small strains and before buckling sets in (Supplementary Fig. 6). Even more, the lateral stretch λ_2 for small aspect ratios is well described by this line, representing the constant Poisson function[2]

$$\tilde{\nu}_{12} = \frac{1 - \lambda_2}{\lambda_1 - 1} = 1/3, \tag{1}$$

over a larger range of longitudinal extension λ_1. By definition of the out-of-plane stretch λ_3 in Equations (3) and (6), the out-of-plane Poisson function $\tilde{\nu}_{13}$ of the analytical model (Equation (7)) takes the value of 0 until buckling sets in. Considered as a planar isotropic material the model thus satisfies the thermodynamic restriction $-1 \leq \nu \leq 0.5$. When considered as an anisotropic three-dimensional material, these restrictions do clearly not apply[3,4], but one finds $\tilde{\nu}_{12} = 1/3$ and $\tilde{\nu}_{13} = 0$ for small strains before buckling.

Supplementary Figures

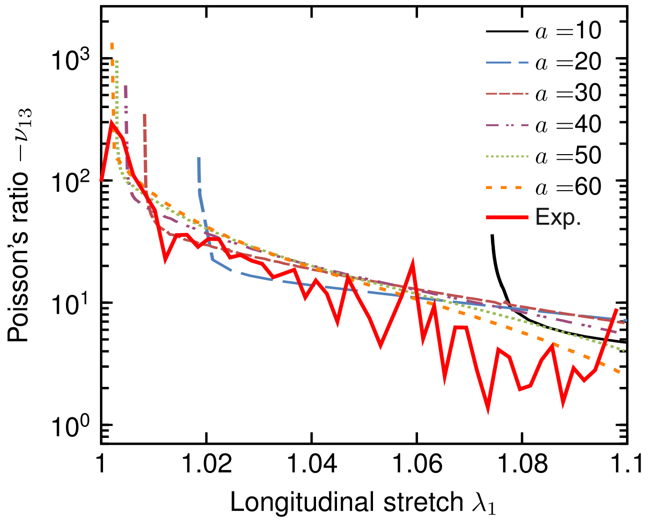

Supplementary Fig. 1: Tangent Poisson's ratios. The out-of-plane tangent Poisson's ratio ν_{13} obtained from the experiments on electrospun PLLA mats (Fig. 2) agrees well with the general trend of those computed with the analytical model (Fig. 1) for a segment aspect ratio $a = 60$. (Experimental data shows the mean calculated from $N = 5$ tensile tests).

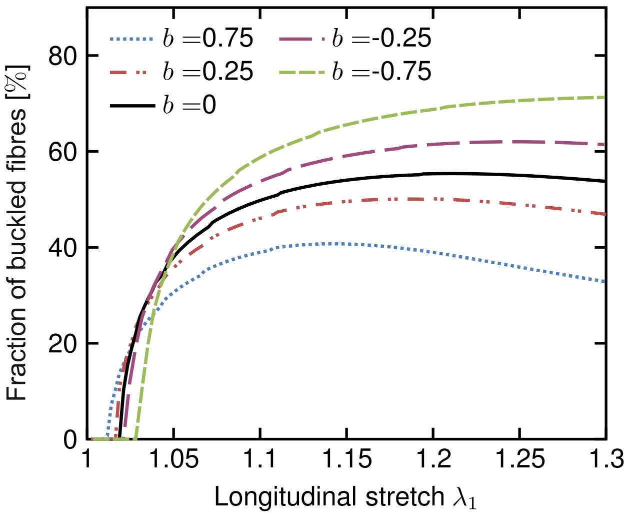

Supplementary Fig. 2: Fraction of buckled segments predicted by analytical model. Fraction of fibres with segment stretch below the critical buckling stretch λ_{cr} for different initial fibre orientation distributions, characterised by the concentration parameter b, and changing with applied tensile stretch λ_1.

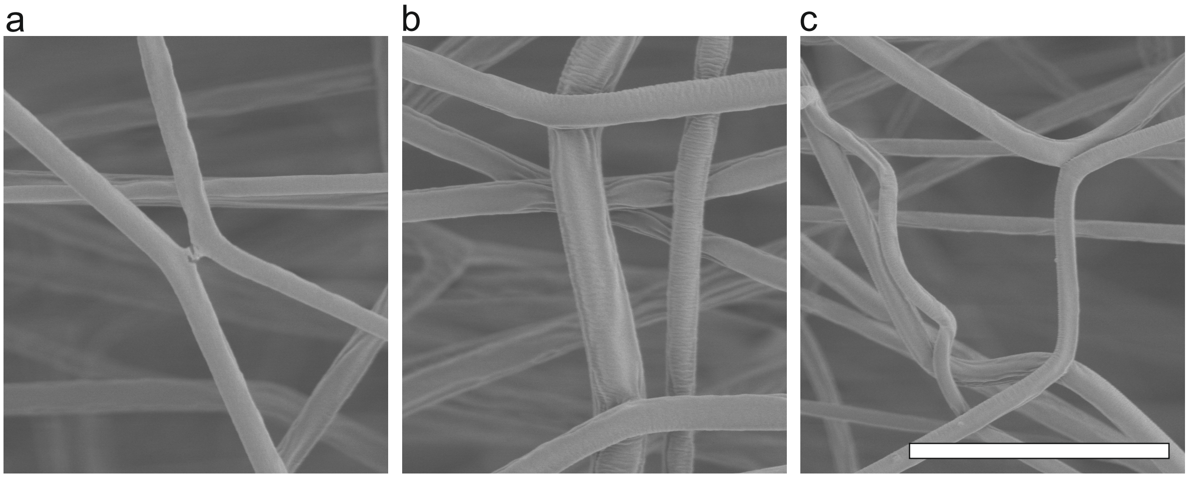

Supplementary Fig. 3: Scanning electron microscopy of connections between fibres. a-c, Exemplary micrographs showing connections between fibres formed in contact regions in the untreated PLLA membrane sample at 10% strain. **a**, Example of a fusion-like connection. Scale bar: 5 µm.

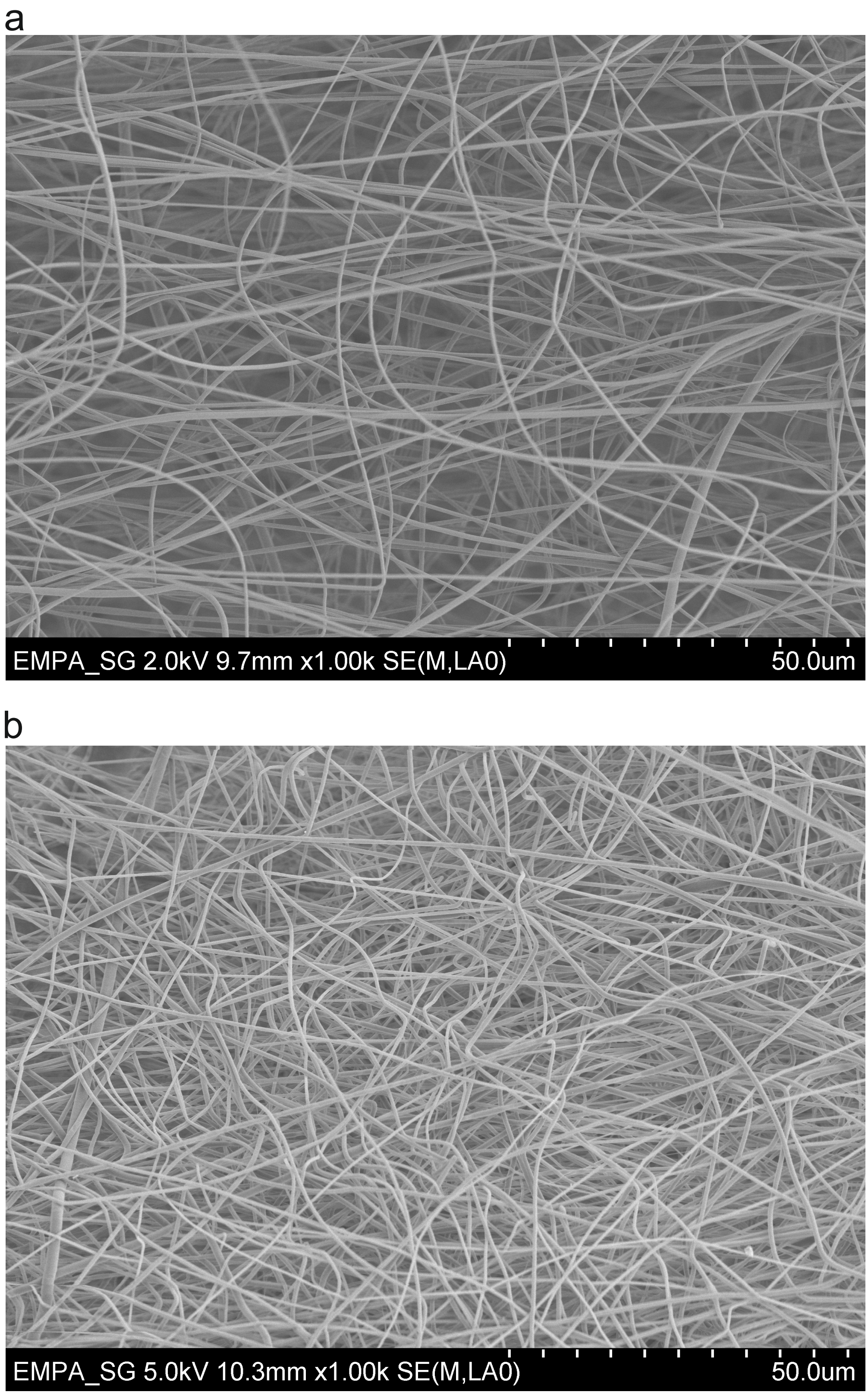

Supplementary Fig. 4: Scanning electron microscopy showing buckled fibre segments before and after treatment with solvent vapour. **a**, Reference network at 10% applied strain. **b**, Post-treated network stretched to 20% applied strain in order to increase the number of buckled segments, in line with the retarded buckling effect predicted by the model (Fig. 1b-e, Supplementary Fig. 1).

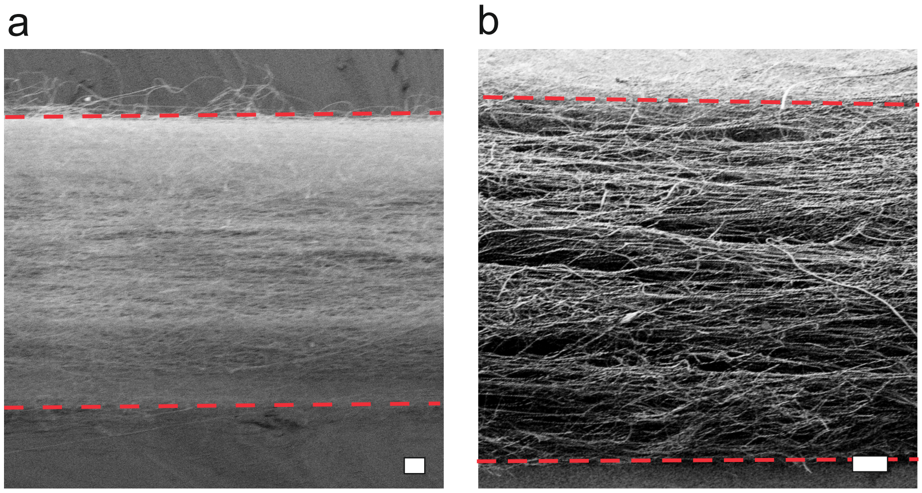

Supplementary Fig. 5: Plastic auxetic deformations. A comparison between side views from in-situ SEM at 10% strain (**a**) and after releasing the applied loads (**b**) reveals that a substantial part of the auxetic expansion remains after unloading. Dashed lines indicate top and bottom edges of the mats. Scale bar: 80 μm.

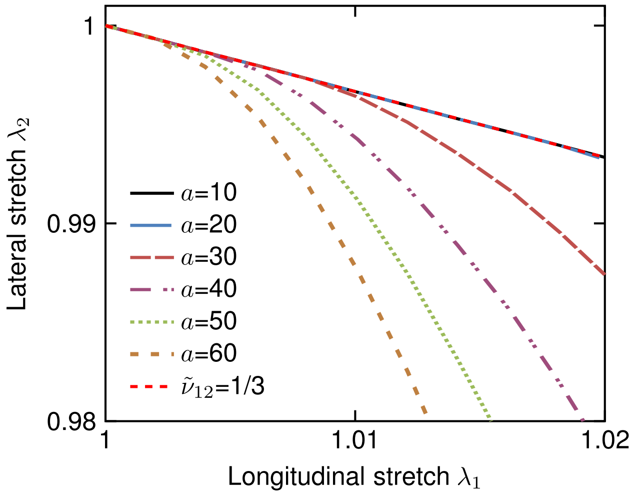

Supplementary Fig. 6: Poisson function of plane rari-constant isotropic material. Close-up view of the lateral contraction λ_2 shown in Fig. 1b plotted together with the Poisson function $\tilde{\nu}_{12} = 1/3$ representative of a planar rari-constant material.

Supplementary References

[1] Vannucci, P. & Desmorat, B. Plane anisotropic rari-constant materials. *Math. Method. Appl. Sci.* **39**, 3271–3281 (2016).

[2] Beatty, M. F. & Stalnaker, D. O. The Poisson function of finite elasticity. *J. Appl. Mech.* **53**, 807–813 (1986).

[3] Vannucci, P. *Anisotropic elasticity* (Springer, 2018).

[4] Ting, T. & Chen, T. Poisson's ratio for anisotropic elastic materials can have no bounds. *Q. J. Mech. Appl. Math.* **58**, 73–82 (2005).
