# Supplementary material for: Random auxetics from buckling fibre networks
Source: Nat Commun. 2019 Oct 25;10:4863. doi: 10.1038/s41467-019-12757-7 (PMC6961253; doi:10.1038/s41467-019-12757-7)
Supplement: Supplementary file 2 — Description of Additional Supplementary Files [file 41467_2019_12757_MOESM2_ESM.docx]

Description of Additional Supplementary Files

**Supplementary Movie 1:** Manual extension of electrospun sample. The manual extension of a 1cm wide strip of an electrospun PLLA mat illustrates the pronounced auxetic behaviour displayed by this material.
